# Diabetes: cost of illness in Norway

**DOI:** 10.1186/1472-6823-10-15

**Published:** 2010-09-20

**Authors:** Oddvar Solli, Trond Jenssen, Ivar S Kristiansen

**Affiliations:** 1 Department of Health Management and Health Economics, P.O. Box 1089 Blindern, N-0317 Oslo, Norway; 2 Rikshospitalet University Hospital, Songsvannsveien 20, N-0027 Oslo, Norway; 3 Institute of Clinical Medicine, Medical Faculty, University of Tromsø, Norway; 4 Institute of Public Health, University of Southern Denmark, DK-5000 Odense, Denmark

## Abstract

**Background:**

Diabetes mellitus places a considerable burden on patients in terms of morbidity and mortality and on society in terms of costs. Costs related to diabetes are expected to increase due to increasing prevalence of type 2 diabetes. The aim of this study was to estimate the health care costs attributable to type 1 and type 2 diabetes in Norway in 2005.

**Methods:**

Data on inpatient hospital services, outpatient clinic visits, physician services, drugs, medical equipment, nutrition guidance, physiotherapy, acupuncture, foot therapy and indirect costs were collected from national registers and responses to a survey of 584 patients with diabetes. The study was performed with a prevalence approach. Uncertainty was explored by means of bootstrapping.

**Results:**

When hospital stays with diabetes as a secondary diagnosis were excluded, the total costs were €293 million, which represents about 1.4% of the total health care expenditure. Pharmaceuticals accounted for €95 million (32%), disability pensions €48 million (16%), medical devices €40 million (14%) and hospital admissions €21 million (7%). Patient expenditures for acupuncture, physiotherapy and foot therapy were many times higher than expenditure for nutritional guidance. Indirect costs (lost production from job absenteeism) accounted for €70.1 million (24% of the €293 million) and included sick leave (€16.7 million), disability support and disability pensions (€48.2 million) and other indirect costs (€5.3 million). If all diabetes related hospital stays are included (primary- and secondary diagnosis) total costs amounts to €535 million, about 2.6% of the total health care expenditure in Norway.

**Conclusions:**

Diabetes represents a considerable burden to society in terms of health care costs and productivity losses.

## Background

Diabetes mellitus places a considerable burden on patients in terms of morbidity [1] and mortality [2] and on society in terms of costs [3-5]. The prevalence of type 2 diabetes is increasing in many countries [6] including Norway [7]. The number of patients with type 1 diabetes has been estimated at 25,000 [8]. In 2005, 117,600 persons in Norway were treated with insulin or oral antidiabetics [9]. We then assume that 92,600 of them have type 2 diabetes. In the Norwegian HUNT study [10] the proportion of patients with type 2 diabetes that was not on antidiabetic pharmaceuticals was 30%. This would imply that the total number of patients with type 2 diabetes is 132,300 (92,600/0.7) [8-11]. Additionally, a large number of persons with type 2 diabetes are assumed to be undiagnosed. It has been estimated that about 3-4% of the population above the age of 30 have type 2 diabetes [8].

Cost-of-illness analysis is a type of study that has been designed to quantify and value all economic consequences of a disease without taking into account the benefits of treatment. Therefore, cost-of-illness analysis in itself may not guide priority setting, but may be useful in designing financing systems and setting priorities for research.

There are two main approaches to cost-of-illness analysis: the prevalence [3,12-15] and the incidence [16] approach. The former accounts for all prevention, treatment and rehabilitation costs incurred during a given year, while the latter measures all such costs for new cases of the disease in a given year (the index year). Future treatment costs are accounted for by estimating the future costs for all individuals who develop the disease in the index year, and the present value of the costs are added to the costs incurred in the index year. The prevalence approach has the advantage of relating to measures of total annual health care expenditure, and it may yield more accurate estimates because it is based, at least in principle, on observed costs rather than projected ones. The advantage of the incidence approach lies in the fact that it provides projections of future costs that may be very different from current ones when incidence is increasing or declining. Such projections, however, may be uncertain.

The aim of this study was to quantify, using the prevalence approach, the societal costs in Norway of type 1 and type 2 diabetes, including indirect costs (productivity losses from diabetes).

## Methods

The study was based on register data for the entire Norwegian population (n = 4.6 million). In addition we performed a survey of 584 persons with diabetes. We aimed at including all diabetes related costs, but some data were unavailable (e.g. depression, erectile dysfunction, neuropathic pain, osteoarthritis, congestive heart failure and pulmonary disease).

### Direct costs

Direct costs are the costs of detection, treatment, prevention, rehabilitation and long-term care arising from an illness. In theory, all relevant health care and non-health care costs are included, but in practice there is a limit to what can be identified and measured. Data were, as far as possible, captured for 2005 and expressed in 2005 EURO (1 € ≈ 8.50 Norwegian Kroner).

#### Inpatient hospital services

From the Norwegian Patient Register (NPR) we obtained information on all hospital stays with the following ICD-10 codes as main or secondary diagnosis: E10 (insulin-dependent diabetes mellitus), E11 (non-insulin-dependent diabetes mellitus), E23.2 (diabetes insipidus), H28.0 (diabetic cataract), N08.3 (glomerular disorders in diabetes mellitus), O24 (diabetes mellitus in pregnancy), P70.0 (syndrome of infant of mother with gestational diabetes), P70.1 (syndrome of infant of a diabetic mother), P70.2 (neonatal diabetes mellitus), R73.0 (abnormal glucose tolerance test) and Z13.1 (special screening examination for diabetes mellitus). For each stay we obtained anonymous data on the primary diagnosis, secondary diagnosis, age, gender, geographic location, length of stay and DRG-weight. In Norway, patients receive a main diagnosis and possibly one or more secondary diagnoses at discharge from hospital. ICD10 has been used since 1999. On the basis of the diagnoses, age, sex and possibly procedures, patients are allocated to a diagnosis related group (DRG). The Directorate of Health performs annual cost studies of a representative sample of hospitals in order to estimate the mean hospital costs of patients in each DRG. Even though the cost estimate may be incorrect for the individual patient, on average they represent reasonable costs for the different types of patients. Hospital services are provided by five Regional Health Authorities, each with an independent board. Regional variation was analysed according to these units.

#### Outpatient clinic visits

Using the same ICD-10 codes as for inpatient services, data on the costs of outpatient clinic visits were provided by The Norwegian Labour and Welfare Administration (NLWA). No data for 2005 were available so data for 2006 were used. The NLWA data encompasses government reimbursements to hospitals. We added the standard patient co-payment per visit (€31). According to the financing model for hospitals, reimbursements and co-payments encompass 40% of the estimated outpatient clinic costs. The sum was therefore adjusted upwards by a factor of 2.5.

#### Physician services

Data on the use of general practitioner (GP) services and private specialists were obtained from NLWA. Claim forms, 90% of which are delivered electronically to NLWA, are provided with ICPC codes. We obtained data on all visits with ICPC codes T89 (insulin-dependent) and T90 (non-insulin-dependent) diabetes. For each patient contact, we obtained data on diagnosis, type of contact, reimbursement and patient co-payment.

#### Drugs

The Norwegian Prescription Registry (NPR) contains information on all prescriptions redeemed from pharmacies. We obtained data for 2005 on the following categories of ATC codes: A10A (insulin and analogues) and A10B (glucose lowering drugs). Additionally, we included the costs of patient reported use of antihypertensive drugs and cholesterol lowering drugs according to a patient survey (see "Other types of health care"). Based on data from the NPR we estimated average costs for one year of treatment with antihypertensive drugs (€154) and cholesterol lowering drugs (€357). This was based on market share of the different drugs available, average dose and prices.

#### Medical equipment

The NLWA keeps account of reimbursement for diabetes self-tests and insulin injection equipment (injection catheters, insulin pens and needles, syringes, lancets for blood sampling). To avoid double counting, the costs of insulin pumps were excluded. Insulin pumps are administered in hospitals and costs are captured in the DRG costing system.

#### Other types of health care

For various other types of resource use where register data are not available, we obtained information through a self-administered questionnaire. A sample of persons with diabetes (n = 1,000) was randomly drawn from the membership file of the Norwegian Diabetes Association (36 000 members in 2006. This file is assumed to encompass most of the individuals in Norway with type 1 diabetes and about 15 000 with type 2 diabetes. We developed a comprehensive questionnaire in collaboration with persons with diabetes and doctors with diabetes care experience. The questionnaire was mailed to the sample patients in May 2007. Non-responders were followed up twice. Finally we had 584 responses that could be used in further analyses. The respondents were asked to state their use of the following types of health care services considered to be relevant among persons with diabetes for the previous three months: physiotherapy, acupuncture, nutrition counselling and GP home visits for hypoglycaemia. They were also asked questions about the duration of the diabetes, type of treatment and occurrence of diabetic related complications. To provide measures of the uncertainty of the estimates we derived confidence intervals by applying bootstrapping, 10 000 draws with replacement. The questionnaire was approved by Regional Committees for Medical Research Ethics and Norwegian Social Science Data Services. Treatment costs were estimated by assigning unit costs to the reported consumption of health care services. Unit costs were taken from professional organizations (physiotherapy, acupuncture) and GP's fee schedule [17].

### Indirect costs

Lacking data on productivity losses from diabetes, we used payments of disability pension and economic support for diabetes related costs as a proxy for indirect costs. Disability pension and economic support are funded by the NLWA. We obtained data on all payments in 2005 for the ICD-10 diagnoses: E10, E11, E23.2, H28.0, H36.0, N08.3, O24, P70.0, P70.1, P70.2, R73.0 and Z13.1. A search was performed with all equivalent ICD-9 codes as well. Furthermore, the following ICPC codes were included: T89 (insulin-dependent diabetes mellitus), T90 (non-insulin-dependent diabetes mellitus), W85 (diabetes during pregnancy) and F83 (retinopathy).

## Results

### Inpatient hospital services

In 2005, there were 8 900 hospital stays with diabetes as the main diagnosis at an estimated total cost of €21 million (Table 1). About 65% of the costs were attributable to insulin-dependent diabetes and 27% to non-insulin-dependent diabetes. Additionally there were 53 000 hospital stays with diabetes as a secondary diagnosis accounting for €242 million in costs. The most frequent main diagnoses when diabetes was a secondary diagnosis were cardiovascular diseases (31% of costs), malignancies (12%) and respiratory diseases (11%). Of the secondary diagnoses, type 2 diabetes (E11) accounted for 65%, while type 1 (E10) accounted for 34%.

**Table 1:** Cost of in-hospital care according to diagnosis

Diabetes as main diagnosis	Number of hospital stays	Total (million €)
Type 1 diabetes*	5 813	13.5
Type 2 diabetes **	2 446	5.7
Other***	625	1.7

Total cost - main diagnosis	8 884	20.9

**Diabetes as secondary diagnosis (ICD-10)**	**Number of hospital stays**	**Total (million €)**

Infections (A00-A99+B00-B99)	1 686	9.3
Malignancies (C00-D89)	5 011	28.6
Neurological diseases (G00-G99)	1 401	4.2
Diseases of the eye (H00-H59)	1 405	2.3
Cardiovascular diseases (I00-I99)	14 545	74.2
Respiratory diseases (J00-J99)	4 504	25.8
Gastrointestinal diseases (K00-K93)	3 423	15.9
Musculoskeletal diseases (M00-M99)	2 777	16.4
Urinary tract diseases (N00-N99)	3 518	16.1
Other***	15 043	49.1

Total cost - secondary diagnosis****	53 313	241.8

* ICD-10 code E10, Insulin-dependent diabetes mellitus

** ICD-10 code E11, Non-insulin-dependent diabetes mellitus

*** ICD-10 codes E23.2, H28, N08.3, O24, P70.0, P70.1, P70.2, R73.0, Z13.1

**** Of the secondary diagnosis type 2 diabetes (E11) accounted for 65 percent, while type 1 (E10) accounted for 34 percent.

The diabetes related in-hospital costs per inhabitant were 27% higher in the geographic region with the highest costs compared to region with the lowest when accounting for admissions with diabetes as main and secondary diagnosis. The total national in-hospital costs would be €302 million if all regions had the same cost level as the most costly, 15% more than the numbers presented in Table 1.

### Outpatient clinic visits

The costs related to outpatient clinic visits in hospitals amounted to €7.9 million (included in Table 2).

**Table 2:** Total cost of diabetes in Norway 2005

	Cost factor	Cost (million €)
Direct costs	In hospital care	20.9
	Outpatient care	7.9
	GP and emergency visits	14.4
	Private practicing specialist services	2.6
	Insulin and analogues (A10A*)	35.1
	Oral glucose lowering drugs (A10B*)	14.5
	Cholesterol lowering drugs	30.7
	Antihypertensive drugs	14.2
	Medical devices	40.1
	Nutritionist guidance	0.8
	Foot therapist	19.4
	Physiotherapy	16.5
	Acupuncture	5.3

	**Subtotal**	(76%) 222.4

Indirect costs	Sickness compensation	16.6
	Permanent disability pension and time limited disability pension	48.2
	Basic and/or supplemental benefits	5.3

	**Subtotal**	(24%) 70.1

	**Total**	292.5

* ATC code

### Physician services

The cost of services from GPs and emergency units was €14.4 million (Table 3) including home visits for hypoglycaemia. The cost relating to private practicing specialists amounted to €2.5 million. On the basis of the survey of persons with diabetes, the estimated annual cost of physician home visits for hypoglycaemia was €0.6 million (Table 4).

**Table 3:** Cost of physician services according to type of contact

	Surgery visits (million €)	Home visits (million €)	Other contacts (million €)	Total cost (million €)
**GPs and emergency units**				
Type 1 diabetes*	0.918	0.0349	0.1633	1.117
Type 2 diabetes **	11.646	0.1600	1.4341	13.240
Other***	0.044	0.0001	0.0104	0.054

Subtotal	12.608	0.1952	1.6078	14.411

**Specialists in private practice**				
Type 1 diabetes****	0.468	0.0000	0.0122	0.480
Type 2 diabetes*****	1.616	0.0007	0.0440	1.661
Retinopathy******	0.156	---	0.0006	0.156
Retinopathy*******	0.180	---	0.0004	0.180
Other********	0.069		0.0012	0.070

Subtotal	2.490	0.0007	0.0582	2.549

Total	15.098	0.1959	1.6660	16.960

* ICPC code T89, Insulin-dependent diabetes

** ICPC code T90, Non-insulin-dependent diabetes

*** ICPC codes F38 Retinopathy and W85 Diabetes during pregnancy

**** ICPC code T89 and ICD-10 code E10, Insulin-dependent diabetes

***** ICPC code T90 and ICD-10 code E11, Non-insulin-dependent diabetes

****** ICPC code F83 Retinopathy

******* ICD-10 code H36.0 Retinopathy

******** E23.2, H28.0, H36.0, N08.3, O24, P70.0, P70.1, P70.2, R73.0, Z13.1

**Table 4:** Costs of various other services*

	Resource use	Unit cost (€) per hour/visit	Cost (million €) (95% CI)**
Type 1 diabetes	Hypoglycaemia - home visit	69	0.3 (0.1 - 0.6)
	Nutritionist guidance	21	0.1 (0.1 - 0.2)
	Foot therapist	53	1.4 (0.9 - 2.1)
	Physiotherapy	28	2.3 (1.2 - 3.4)
	Acupuncture	35	0.4 (0.1 - 0.8)

Subtotal	-----		4.5

Type 2 diabetes	Hypoglycaemia - home visit	69	0.3 (0.0 - 0.4)
	Nutritionist guidance	21	0.8 (0.5 - 1.2)
	Foot therapist	53	19.4 (17.5 - 22.0)
	Physiotherapy	28	16.5 (11.3 - 21.3)
	Acupuncture	35	5.3 (2.6 - 10.6)

Subtotal	-----		42.3

Total			46.8

* Data collected in the patient survey

** Confidence intervals based on bootstrapping

### Drugs

The cost of hypoglycaemic agents for treating diabetes was €49.6 million (17% of total costs) (Table 5) of which €35.1 million (70%) represented insulin and analogues (A10A) and the rest oral glucose lowering drugs (A10B). Within the insulin group the cost of intermediate-acting insulin (A10AC) was €15 million and fast-acting insulin (A10AB) was €12.5 million. In the group of glucose lowering drugs (A10B) the cost of sulphonamides, urea derivatives (A10BB) was €6.2 million and biguanides (A10BA) €4.7 million. For antihypertensive drugs the estimated cost was €1.2 and €13.0 million for type 1 and 2 diabetes, respectively, while it was €2.4 and €28.3 for cholesterol lowering drugs.

**Table 5:** Cost of hypoglycaemic agents, cholesterol lowering drugs and antihypertensive drugs

	Number of users	Million DDD	Costs* (million €)
Insulin and analogues (A10A**)***	47 073	28.9	35.1

Oral glucose lowering drugs (A10B**)***	85 014	36.2	14.5

Cholesterol lowering drugs****	85 880	---	30.7

Antihypertensive drugs****	92 172	---	14.2
Total	---	---	94.5

* Costs in terms of prices in Pharmacy sales prices including VAT

** ATC code

*** Data from the National prescription database

**** Data from a patient survey

### Medical equipment

Expenditure on diabetes related medical equipment was €40 million (Table 6). The largest component here was glucose tests accounting for €32 million (80% of the total). Lancets for blood sampling accounted for approximately €5.3 million, (13% of the total).

**Table 6:** Cost of medical devices

	Reimbursement	Patient co-payment	Total Costs (million €)
Glucose tests	29.484	2.096	31.580
Lancets for blood sampling	4.885	0.378	5.262
Injection catheter	0.01	0.00024	0.010
Insulin pens	0.44	0.036	0.476
Needles for insulin pens	2.394	0,182	2.576
Insulin syringe	0.105	0.008	0.113
Urine test sticks	0.040	0.003	0.043

Total	37.358	2.703	40.061

### Other types of costs

Among costs estimated on the basis of the patient survey (Table 4), physiotherapy accounted for €18.8 million, foot therapy €20.8 million, acupuncture €5.7 million and nutrition guidance €0.9 million.

### Indirect costs

The costs related to sick leave were €16.7 million (Table 7) of which type 2 diabetes accounted for 85%. Total costs related to time limited disability support and disability pensions amounted to €48.2 million (Table 8), of which disability pensions accounted for €46.4 million (96%). Cost of basic and supplemental benefits was €5.3 million (Table 9).

**Table 7:** Cost of sick leave due to diabetes

Diagnose (ICPC)	Number of patients	Days of support	Payment (million €)
Insulin-dependent (T89)	269	17 602	1.6
Non-insulin-dependent (T90)	2 593	156 706	14.1
Retinopathy (F83)	149	7 948	0.8
Diabetes during pregnancy (W85)	38	1 523	0.13

Total	3049	183 779	16.7

**Table 8:** Cost of disability pension and time limited disability pension related to diabetes

	Benefit (€)	Number of patients	Expenditure (million €)
Time limited disability support	17 250	112	1.9
Disability pension	16 689	2 775	46.4

Total	16 711	2 887	48.2

**Table 9:** Basic and supplemental benefits related to diabetes*

	Number of patients	Expenditure (million €)
Basic benefits	1058	1.4
Supplemental benefits	1876	3.9

Total	2934	5.3

* Approximately 15% of overall receivers of basic and supplemental benefits are lacking diagnosis in the database

### Total costs

Total costs were €293 million (Table 2) when hospital stays with diabetes as secondary diagnoses were excluded and €535 million when they were included. The largest component was medicines with €95 million (32% of the total). The second largest was disability pensions with €48 million (16%). Medical devices contributed €40 million (14%) and hospital admissions €21 million (7%).

## Discussion

The results of this study clearly indicate that diabetes places a financial burden on the persons with diabetes themselves and furthermore the Norwegian public health care system. The total costs of treating diabetes in Norway in 2005 amounted to about €293 million or 1.4% of total health care expenditures [18], or 2.6% if all diabetes related hospital stays are included. Interestingly, patient expenditures for acupuncture, physiotherapy and foot therapy were many times that of those for nutritional guidance. In addition, diabetes imposes costs on society in terms of lost production from job absenteeism and premature mortality.

Cost-of-illness analyses in general should always be viewed in the context of potential limitations: some costs may be underestimated, some costs may be overestimated and some costs are omitted. Regarding our study, we have not accounted for productivity losses from diabetes-related premature mortality because we adopted the prevalence approach. In addition, diabetes may cause complications such as cardiovascular disease, renal failure, retinopathy, erectile dysfunction and others that incur costs. To the extent diabetes is stated as a secondary diagnosis at hospital discharge, such costs are included in the €535 million estimate. With respect to some of the complications, there are many causal factors and no reliable data on the fraction attributable to diabetes.

When estimating the cost of in-hospital care on the basis of the main diagnosis (Table 1), some hospitals stays may be lost even when diabetes was the main cause of the stay. Because hospitals in Norway have partial DRG financing, the choice of primary diagnosis may be influenced by the financial consequences of choice of primary diagnosis. The results of a Norwegian study [19] indicate that diabetes patients tend to have higher costs than the average patient within certain DRGs. To the extent that this is the case, our estimates are biased. A lack of diabetes diagnosis may also bias costs related to disability pensions and sick leave, physician visits, outpatient clinic visits and certain other types of services where the costing is based on diagnosis. Finally, costs based on patient reported use of care may be underestimated because patients do not recall all use of care.

When including in-hospital care for stays where diabetes was a secondary diagnosis (Table 1), some stays may not be caused in full by diabetes. If for example diabetes is stated as secondary diagnosis for a patient discharged from hospital because of a malignant disease, at most a minority of the costs may be attributable to diabetes.

Costs of hypoglycaemic agents stems from the national prescription database and contain all prescriptions redeemed in pharmacies. Costs related to drugs provided in hospitals are included in the DRG reimbursement to hospitals. Pharmaceuticals used to prevent or treat diabetes related complications are difficult to quantify, but lipid lowering and antihypertensives are included on the basis of the patient survey.

The NLWA keeps account of reimbursement for diabetes related medical devices and these costs are likely to be complete. Also, drug costs are quite accurate because all pharmacies register prescriptions electronically and transfer their data to the central registry.

We have included some types of costs that we consider relevant for persons with diabetes, such as doctor home visits related to hypoglycaemia, nutritionist guidance, foot therapy, physiotherapy and acupuncture. We can not attribute all costs gathered in the patient survey to diabetes. For example, there are reasons other than diabetes for having acupuncture. It should be noted that the survey we undertook may not be entirely representative of the diabetes population in Norway, especially for type 2 diabetes. Some cost estimates (GP home visits for hypoglycaemia, foot therapy, nutritional guidance, physiotherapy, acupuncture, costs of cholesterol lowering- and antihypertensive drugs) may consequently be biased, but the impact on any bias will be small because the relevant costs were small.

Our study provides some important general lessons about the cost structure of diabetes care. First, the main direct cost-drivers from diabetes are hospital services, pharmaceuticals and medical devices. These services are reimbursed in part or in full by governments in most industrialised countries. Second, other types of services such as foot therapy, physiotherapy, and acupuncture may represent considerable costs, but often receive only partial and sometimes no reimbursement by governments. In Norway, the use of foot therapy is paid in full by the patient, while in contrast the cost of foot ulcer treatment and amputations is covered almost fully by the government. This may seem paradoxical as untreated foot ulcers may lead to infections and ultimately amputation. Finally, the study reveals a high level of spending on acupuncture compared to much lower spending on nutritional guidance. Given the importance of diet for the progress of the disease, this result is somewhat paradoxical and suggests that patients could benefit from a different spending pattern.

We found that hospital costs with diabetes as main diagnosis were twice as high for type 1 diabetes as for type 2. However, a large proportion of those with non-insulin-dependent diabetes had CVD as the primary diagnosis. Diabetes is likely an important causal factor for CVD among these patients which indicates that type 2 diabetes still is a major cost driver. It is therefore likely that type 2 diabetes is more important than type 1 diabetes with respect to hospital costs.

The number of individuals on oral glucose lowering drugs was almost twice the number of users of insulin and analogues. In terms of costs, the pattern was opposite in that the total cost of insulin and analogues was twice the cost of oral glucose lowering drugs. This indicates that treatment of type 2 diabetes becomes more costly with disease progression because insulin is increasingly prescribed with progression.

Our results are somewhat different from those reported elsewhere. In Sweden the estimated costs of hypoglycaemia related to type 2 diabetes was €14.10 per patient per year [20] while our data would suggest about €3 per patient. The Swedish costs are higher because of a higher reported prevalence of hypoglycaemia and the inclusion of indirect costs.

One should be aware of methodological differences when comparing the results of cost-of-illness analyses. We used a prevalence approach; studies relying on an incidence approach with prediction of future costs may yield higher values. Also, the method for valuing absence from productive work may have considerable impact on the results of cost-of-diabetes studies. Clearly, the more types of diabetes related costs that are included, the higher the estimated costs. A recent review [21] suggests that there is a general tendency for indirect costs to make up a slightly larger proportion of total costs than direct costs. In the studies reviewed, the proportion of indirect costs was in the range of 25-64%.

A study performed in Ireland [22] estimated that the costs of treating diagnosed type 2 diabetes was 4.1% of the total health care expenditure. Hospitalisations accounted for almost half of overall costs, while ambulatory and medicines costs accounted for 27% and 25%.

In an early Swedish [3] study, the costs of diabetes amounted to 5.7 billion Swedish Kroner (SEK) (€570 million) of which 43% represented direct costs. Hospital care estimates were based on the main diagnosis and represented the main component of direct costs. The distribution among the different types of direct costs were about the same in the Swedish study as our. The indirect costs in the Swedish study represented 57% of the total compared to 24% in our study when including only hospital admissions with diabetes as the main diagnosis. This difference is in part attributable to the fact the Swedish study included productivity losses caused by premature mortality while ours did not. Whether the remaining difference between the two studies is attributable to difference in time or difference in real costs is unclear.

In a recent Swedish study [23] which report increasing costs of diabetes over time, another approach to COI analysis is used. Diabetes prevalence and attributable risks for diabetes complications were used to estimate the diabetes-related costs. This approach should result in an estimate of the COI that is between estimates based on diabetes as the primary diagnosis and estimates based on diabetes as the primary as well as the secondary diagnosis.

The wide variation in methodology makes comparison of the results difficult and calls for standardisation of methods. Patient organisations might play a role in developing guidelines for COI studies. Additionally, there is a need for more research into how choice of methods impact the results using data from the same country and the same time rather than comparing across countries. Even though COI represent a basis for allocating research resources, most research should be directed at studies of intervention effectiveness and how care can be provided in the most efficient way. The latter in practice means cost-effectiveness studies, and our COI study could be used as a toolbox for analysts in need of cost data. If later studies are performed in the same way, it may provide useful insight in how costs develop over time.

## Conclusions

In conclusion, the cost of diabetes represents 1.4% - 2.6% of the total health care expenditures in Norway, depending on how diabetes related hospitalisation is accounted for. The high diabetes costs indicate that society may do well in devoting resources to diabetes prevention and research.

## Competing interests

Trond Jenssen and Ivar Sønbø Kristiansen have received honoraria from various pharmaceutical companies that market drugs that may be used by persons with diabetes. Trond Jenssen is medical advisor to the Norwegian Diabetes Association.

## Authors' contributions

OS (lead author) developed the study design, collected data, performed the analyses, drafted the manuscript and approved the final version. TJ provided inputs on design, revised the manuscript during the writing and approved the final version. ISK provided inputs on design, revised the manuscript during the writing and approved the final version.

## Pre-publication history

The pre-publication history for this paper can be accessed here:

http://www.biomedcentral.com/1472-6823/10/15/prepub

## References

[B1] UK Prospective Diabetes Study Group Tight blood pressure control and risk of macrovascular and microvascular complications in type 2 diabetes: UKPDS 38 BMJ 1998 10 703 713 9732337 PMC28659

[B2] Stamler J Vaccaro O Neaton JD Wentworth D Diabetes, other risk factors, and 12-yr cardiovascular mortality for men screened in the Multiple Risk Factor Intervention Trial Diabetes Care 1993 10 434 444 10.2337/diacare.16.2.434 8432214

[B3] Henriksson F Jonsson B Diabetes: the cost of illness in Sweden Journal of Internal Medicine 1998 10 461 468 10.1111/j.1365-2796.1998.00388.x 9893099

[B4] Henriksson F Agardh CD Berne C Bolinder J Lonnqvist F Stenstrom P Ostenson CG Jonsson B Direct medical costs for patients with type 2 diabetes in Sweden Journal of Internal Medicine 2000 10 387 396 10.1046/j.1365-2796.2000.00749.x 11123503

[B5] Jonsson B Diabetes--the cost of illness and the cost of control. An estimate for Sweden 1978 Acta Med Scand Suppl 1983 10 19 27 6576620 10.1111/j.0954-6820.1983.tb08543.x

[B6] Wild S Roglic G Green A Sicree R King H Global Prevalence of Diabetes: Estimates for the year 2000 and projections for 2030 Diabetes Care 2004 10 1047 1053 10.2337/diacare.27.5.1047 15111519

[B7] Midthjell K Kruger O Holmen J Tverdal A Claudi T Bjorndal A Magnus P Rapid changes in the prevalence of obesity and known diabetes in an adult Norwegian population. The Nord-Trondelag Health Surveys: 1984-1986 and 1995-1997 Diabetes Care 1999 10 1813 1820 10.2337/diacare.22.11.1813 10546013

[B8] Stene LC Midthjell K Jenum AK Skeie S Birkeland KI Lund E Joner G Tell GS Schirmer H [Prevalence of diabetes mellitus in Norway] Tidsskr Nor Laegeforen 2004 10 1511 1514 15195154

[B9] The Norwegian Prescription Database http://www.norpd.no

[B10] Midthjell K [Diabetes in adults in Nord-Trøndelag. Epidemiological and public health aspects of diabetes mellitus in a large, non-selected Norwegian population] Phd-Thesis 2001 The Norwegian University of Science and Technology (NTNU)

[B11] Strom H Engeland A Eriksen E Sakshaug S Ronning M [How many and who are receiving medication for diabetes mellitus?] Tidsskr Nor Laegeforen 2006 10 768 770 16541171

[B12] American Diabetes Association Economic Costs of Diabetes in the U.S. in 2002 Diabetes Care 2003 10 917 932 10.2337/diacare.26.3.917 12610059

[B13] American Diabetes Association Economic Costs of Diabetes in the U.S. in 2007 Diabetes Care 2008 10 596 615 10.2337/dc08-9017 18308683

[B14] Dawson KG Gomes D Gerstein H Blanchard JF Kahler KH The Economic Cost of Diabetes in Canada, 1998 Diabetes Care 2002 10 1303 1307 10.2337/diacare.25.8.1303 12145225

[B15] Koster I von Ferber L Ihle P Schubert I Hauner H The cost burden of diabetes mellitus: the evidence from Germany - the CoDiM Study Diabetologia 2006 10 1498 1504 10.1007/s00125-006-0277-5 16752168

[B16] Hart WM Espinosa C Rovira J A simulation model of the cost of the incidence of IDDM in Spain Diabetologia 1997 10 311 318 10.1007/s001250050680 9084970

[B17] Fee Schedule for general practice (in Norwegian: Fastlegetariffen, Normaltariff). 2005 2005 Norwegian Medical Assosiation, Oslo

[B18] Statistics Norway 2008 http://www.ssb.no/helsesat/arkiv/art-2006-05-30-01.html

[B19] SINTEF 2008 http://www.sintef.no/project/Samdata/rapporter/Sykehusbruk_blant_eldre_i_Skandinavia_i_2002_rapport_STF78_A055015.pdf

[B20] Jonsson L Bolinder B Lundkvist J Cost of hypoglycemia in patients with Type 2 diabetes in Sweden Value Health 2006 10 193 198 10.1111/j.1524-4733.2006.00100.x 16689714

[B21] Ettaro L Songer TJ Zhang P Engelgau MM Cost-of-illness studies in diabetes mellitus PHARMACOECONOMICS 2004 10 149 164 10.2165/00019053-200422030-00002 14871163

[B22] Nolan JJ O'Halloran D McKenna TJ Firth R Redmond S The cost of treating type 2 diabetes (CODEIRE) Ir Med J 2006 10 307 310 17274175

[B23] Bolin K Gip C Mörk A-C Lindgren B Diabetes, healthcare cost and loss of productivity in 1987 and 2005 - a register-based approach Diabetic Medicine 2009 10 928 934 10.1111/j.1464-5491.2009.02786.x 19719715

